# High prevalence of Wilms tumor 1 expression in multiple myeloma and plasmacytoma: A cohort of 142 Asian patients’ samples

**DOI:** 10.3389/pore.2023.1610844

**Published:** 2023-01-24

**Authors:** Ployploen Phikulsod, Sanya Sukpanichnant, Chutima Kunacheewa, Thaweesak Chieochansin, Mutita Junking, Pa-Thai Yenchitsomanus

**Affiliations:** ^1^ Siriraj Center of Research Excellence for Cancer Immunotherapy (SiCORE-CIT), Division of Molecular Medicine, Research Department, Faculty of Medicine Siriraj Hospital, Mahidol University, Bangkok, Thailand; ^2^ International Graduate Program in Immunology, Department of Immunology, Faculty of Medicine Siriraj Hospital, Mahidol University, Bangkok, Thailand; ^3^ Division of Hematology, Department of Medicine, Faculty of Medicine Siriraj Hospital, Mahidol University, Bangkok, Thailand; ^4^ Department of Pathology, Faculty of Medicine Siriraj Hospital, Mahidol University, Bangkok, Thailand

**Keywords:** multiple myeloma, immunohistochemistry (IHC), plasmacytoma, WT1 protein, cohort study Thailand, Asian cohort, treatment outcome, clinical relevance

## Abstract

Wilms tumor 1 (WT1) is a promising target antigen for cancer immunotherapy. However, WT1 protein expression and its clinical correlation in multiple myeloma (MM) patients are still limited. We, therefore, investigated WT1 expression in 142 bone marrow and plasmacytoma samples of MM patients at different stages of the disease by immunohistochemistry. The correlations between WT1 expression and clinical parameters or treatment outcomes were evaluated. The overall positive rate of WT1 expression was 91.5%; this high prevalence was found in both bone marrow and plasmacytoma samples, regardless of the disease status. Cytoplasmic WT1 expression was correlated with high serum free light chain ratio at presentation. However, no significant association between WT1 expression and treatment outcome was observed. This study confirms the high prevalence of WT1 expression in an Asian cohort of MM, encouraging the development of immunotherapy targeting WT1 in MM patients, particularly in those with extramedullary plasmacytoma or relapsed disease.

## Introduction

Wilms tumor 1 (WT1) is a zinc-finger transcription factor encoded by *WT1* gene on human chromosome 11p13 (1). WT1 is overexpressed in many cancers and is correlated with poor prognosis in some types of cancers (2–6). WT1 was ranked as the most promising target antigen for cancer immunotherapy in 2009 by the US National Cancer Institute (7). Since then, immunotherapy targeting WT1 has been studied and clinical efficacies were shown in various cancer models (6).

Multiple myeloma (MM) is the second most common hematologic malignancy worldwide (8). The median age at the diagnosis is 66–70 years of age. Even though the treatment of MM has been improved and able to increase the overall survival of the patients, MM is still considered incurable (9). Targeted therapy and immunotherapies have been developed and approved for the treatment of MM (9). A cancer vaccine is an alternative method that could deepen disease responses after the failure of conventional treatment in patients with relapsed disease. Many target antigens, including WT1, have been studied in MM patients (10). At an early stage of the studies, WT1 seemed not to be a potential target for MM because of its low RNA expression in bone marrow samples (2,11). Despite that, myeloma cells were found to be highly sensitive to lysis by WT1-specific cytotoxic T lymphocytes (12). WT1-specific T cells were also found in the MM patients and their increment correlated with disease control after donor lymphocyte infusions (13). Moreover, clinical efficacies of WT1 vaccine in MM were shown in clinical trials (14,15). This led to the fast-track approval of WT1 peptide vaccine as maintenance for high-risk MM by US FDA in 2018.

Previously, WT1 protein was found to be expressed in all 15 MM patients in the western population using immunohistochemistry (IHC) method (13). Therefore, instead of detection of mRNA expression, WT1 immunostaining could be more useful as a marker for immunotherapy targeting WT1 in MM. However, the data on WT1 protein expression in MM is still limited and controversial. Another study from China showed that WT1 IHC staining was positive only in 30% of MM samples (*n* = 62) (16). To study the prevalence of WT1 protein expression in MM, we used IHC method to detect WT1 protein expression in a larger and more variety of MM samples in Thai patients as a representative of the Asian cohort.

## Materials and methods

### Tissue samples and associated clinical data

Samples from patients who has been diagnosed with MM at Siriraj Hospital between January 2014 and December 2016 were included. To verify the diagnosis, diagnostic reports along with hematoxylin and eosin slides were reviewed. Cases with inadequate paraffin-embedded samples and less than 20% of myeloma cell involvement were excluded. Associated clinical data were retrieved from electronic medical records. Detailed baseline clinical characteristics were listed in Table 1.

**TABLE 1:** Clinicopathological features of multiple myeloma patients.

Clinical factors	Overall (*n* = 142)	WT+ (*n* = 130, 91.5%)	WT- (*n* = 12, 8.5%)	*p*-value
Age (years)	62 ± 10	62.4 ± 10.6	61.4 ± 9.3	0.760
Gender, % (n)				1.000
Female	50% (71)	50% (65)	50% (6)	
Subtype of MM, % (n)				0.914
IgG	54.8%	54.6%	75%	
IgD	1.4%	0.8%	0.8%	
IgA	13.9%	14.6%	8.3%	
Light chain	25%	26.2%	16.7%	
Heavy chain	0.7%	0.8%	0%	
Isolated plasmacytoma	2.1%	1.5%	0%	
NA	1.4%	1.5%	0%	
ISS staging, % (n)				0.778
Stage I	7.2% (5)	7.8% (5)	0% (0)	
Stage II	31.9% (22)	31.3% (20)	40% (2)	
Stage III	60.9% (42)	60.9% (39)	60% (3)	
Clinical manifestation				
Hemoglobin (g/dL)	8.8 ± 2.1	8.8 ± 2.2	8.6 ± 2	0.815
Platelets (10^3^cells/uL)	182 (32–561)	178 (32–561)	224 (73–366)	0.783
Creatinine (mg/dL)	1.2 (0.4–15.4)	1.2 (0.4–15.4)	1.2 (0.5–2.3)	0.513
Corrected Ca^2+^ (mg/dL)	10.4 ± 1.7	10.4 ± 1.7	10.6 ± 1.2	0.788
Beta-2-microglobulin (mg/dL)	6.6 (1.8–69.1)	6.7 (1.8–69.1)	6.3 (2.9–12.5)	0.438
M protein (g/dL)	4.04 ± 1.98	4.01 ± 2.01	4.41 ± 1.67	0.704
Serum free light chain ratio	94.2 (1.1–5,537.9)	108.5 (1.1–5,537.9)	8.02 (5.6–241.7)	0.084
Bone fracture, % (n)	43.2% (41/95)	41.6% (37/89)	66.7% (4/6)	0.230
Status of disease, % (n)				1.000
First diagnosis	64.8% (92)	64.6% (84)	66.7% (8)	
Relapse	35.2% (50)	35.4% (46)	33.3% (4)	
Tissues, % (n)				0.704
Bone marrow	80.3% (114)	80.8% (105)	75% (9)	
Plasmacytoma	19.7% (28)	19.2% (25)	25% (3)	
Complete remission rate, % (n)	45.8% (27/59)	49.2% (29/54)	40% (2/5)	1.000
Novel therapy, % (n)	69% (49/71)	68.2% (45/66)	80% (4/5)	1.000
Transplantation rate, % (n)	17.7% (14/79)	17.6% (13/74)	20% (1/5)	1.000

### Immunohistochemistry

Tissue sections of 3 µm thickness were prepared. The tissue sections were retrieved at 95°C, pH 8.5, for 64 min in CC1 solution (Ventana). Non-specific activities were blocked with 3% H_2_O_2_ and antibody diluent (Ventana). Prediluted 1:500 mouse anti-WT1 antibody (clone 6F-H2; Cell Marque) was incubated for 1 h at 36°C. A positive signal was detected using the amplification and UltraView Universal DAB detection kit (Roche). Sections from Wilms tumor, kidney, tonsil, and samples with omission of the primary antibody as positive and negative controls.

### Evaluation method

The proportion of positive myeloma cells and reaction strength for WT1 protein expression were determined. The level and distribution of expression were reviewed and estimated in agreement by three investigators. The positive reaction strength was described as −, +, + and +++. To assess the extent of immunoreactivity, H-score was calculated by the formula: (3 x % strongly staining cells) + (2 x % moderately staining cells) + % weakly staining cells.

### Statistical analysis

Statistical analyses were carried out using SPSS 13.0 for Windows (SPSS, USA). Categorical data are given as numbers and percentages, and continuous data are reported as either mean ± standard deviation (SD) (normal distribution) or median and range (non-normal distribution). In the univariate analysis of the independent samples, *t*-test was used for normally distributed variables, and the Mann-Whitney U test was used for non-normally distributed variables. Pearson’s X^2^ or Fisher’s exact test was used to examine the association between categorical variables. Correlation among the factors was calculated using Spearman/Pearson correlation coefficient test. Patient survival was analyzed using the Kaplan-Meier method and a log-rank test. A two-sided *p*-value of less than 0.05 was considered statistically significant.

## Results

### Baseline patient characteristics and specimens

A total of 142 specimens from 95 MM patients were studied. The average age of the patients was 62 years. Half of them were females. The majority of patients had IgG subtype. Ninety-two samples (65%) were collected at diagnosis whereas 50 samples (35%) were collected at the time of relapse. One hundred and fourteen samples (80%) were bone marrow tissues and twenty-eight (20%) were plasmacytoma tissues. For the plasmacytoma specimens (*n* = 28), half of them arose from bone and the other half were hematogenous spreading of plasma cells. The clinical characteristics and laboratory findings of the patients are summarized in Table 1. There were no significant differences in baseline characteristics between the cases with positive WT1 staining and those with negative WT1 staining.

### WT1 protein expression

**FIGURE 1:**
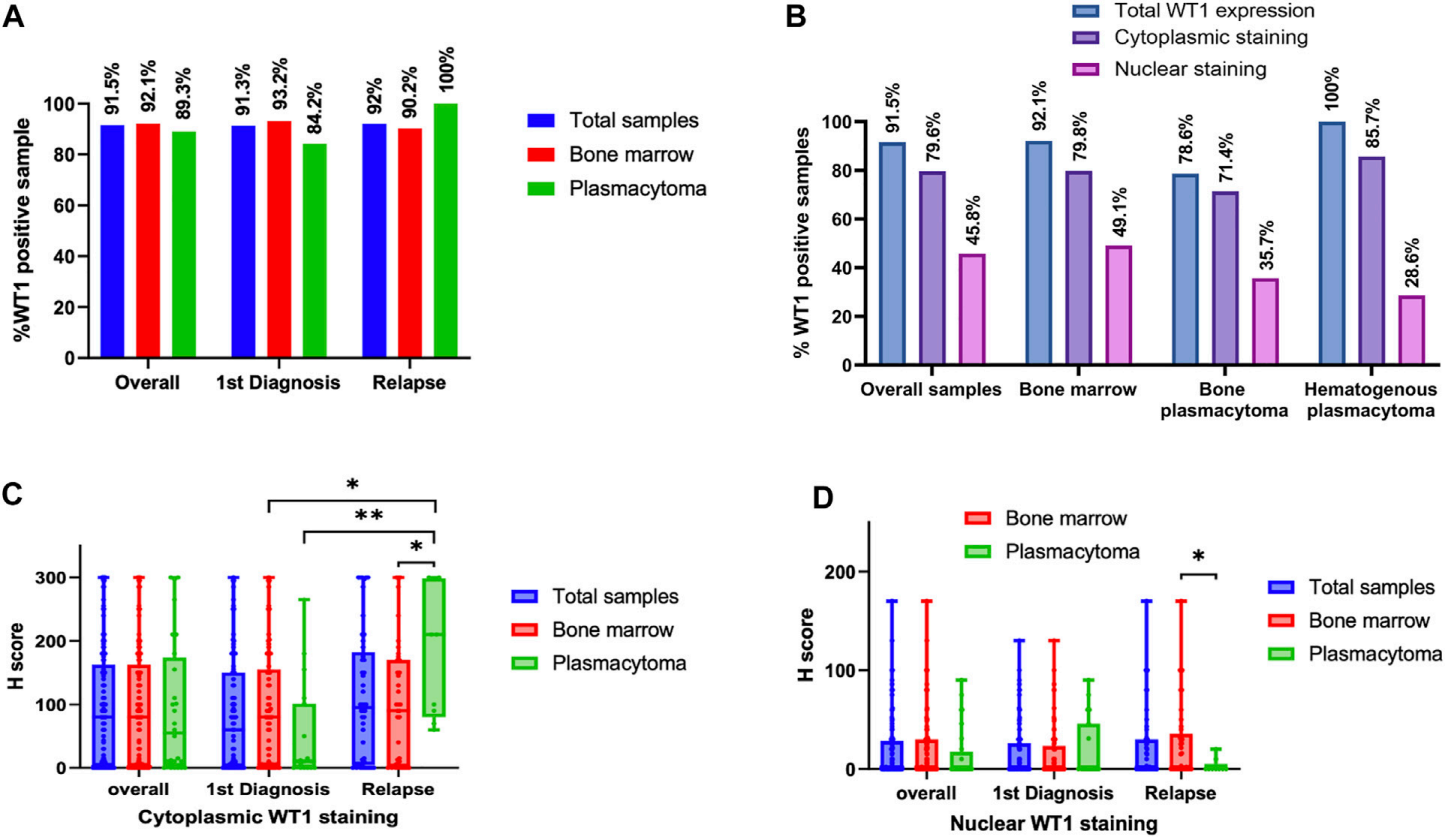
WT1 protein expression in tissues at different stages of multiple myeloma (MM): Rates of WT1 protein expression in MM tissues at different clinical settings **(A)**, Rates of cytoplasmic and nuclear WT1 staining in different tissue samples **(B)**, Histology score (H-score) of WT1 cytoplasmic staining **(C)**, and nuclear staining **(D)** in different myeloma samples at first diagnosis or relapse stage (*p < 0.05, **p < 0.01).

**FIGURE 2:**
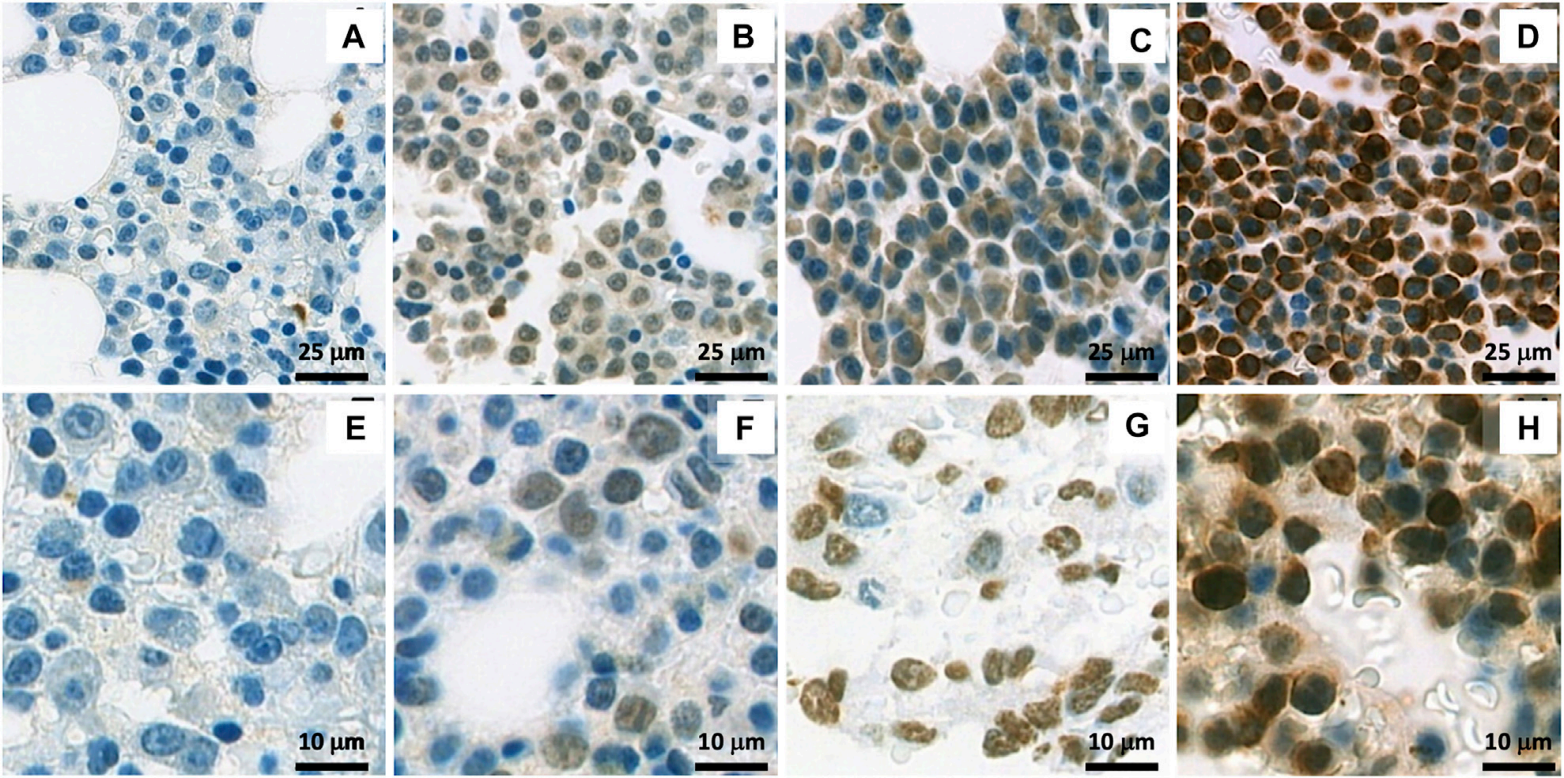
Immunohistochemistry (IHC) staining of WT1 protein: **(A–D)** show a representative cytoplasmic staining with different intensities in samples: Negative staining **(A)**, + **(B)**, ++ **(C)** and +++ **(D)**; **(E–H)** show a representative nuclear staining with different intensities in samples: Negative staining **(E)**, + **(F)**, ++ **(G)** and +++ **(H)**.

WT1 protein expression was found in 91.5% of total samples. The rates of WT1 expressions were 91.3%, 92.0%, 92.1%, and 89.3% in samples at the diagnosis, at relapse, bone marrow, and plasmacytoma, respectively (Figure 1A). In the plasmacytoma samples (*n* = 28), soft-tissue plasmacytoma (*n* = 14) and relapsed plasmacytoma (*n* = 9) had an exceptional high rate of WT1 protein expression (100%) as compared to bone plasmacytoma (78.6%, *n* = 11/14) (*p* = 0.067) (Figure 1B). Both cytoplasmic staining and nuclear staining of WT1 were observed (Figure 2). WT1 cytoplasmic staining was detected in 79.6% (114/142) of samples with an H-score ranging from 0 to 300, and a median score of 80, whereas nuclear staining was detected in 45.8% (65/142) of samples and H score ranged from 0 to 170, with a median score of 0 (Figure 1B; Table 2). WT1 positive cells per sample in the samples at a relapse stage were higher than that of the samples at the first diagnosis (50% vs. 30%) but without a statistical significance (*p* = 0.222). There was no difference in the overall rate and intensity of WT1 positivity between myeloma cells in the bone marrow and extramedullary samples. However, in a subgroup analysis, the highest cytoplasmic WT1 expression was found in relapsed plasmacytoma samples (Figure 1C). A median WT1 cytoplasmic H score in relapsed plasmacytomas was 210 (60–300) in comparison to 10 (0–265) (*p* = 0.002), 80 (0–300) (*p* = 0.011), and 90 (0–300) (*p* = 0.031) in plasmacytoma samples at first diagnosis, bone marrow samples at first diagnosis, and bone marrow samples at relapse, respectively (Figure 1C; table 2). The median percentage of cytoplasmic positive cells per sample was 99% (30–100) in the relapsed plasmacytoma compared to 5% (0–100) (*p* = 0.004), 30% (0–100) (*p* = 0.016), and 40% (0–100) (*p* = 0.023) in plasmacytoma samples at first diagnosis, bone marrow samples at first diagnosis, and bone marrow samples at relapse, respectively. The comprehensive data of WT1 staining was shown in Figure 1 and Table 2.

**TABLE 2:** Characteristics of WT1 positivity in samples.

Samples	Cytoplasmic staining			Nuclear staining		
	% Positive samples (n)	% Positive cells, median (range)	IHC score median (range)	% Positive samples (n)	% Positive cells, median (range)	IHC score median (range)
Overall	79.6 (113)	30 (0–100)	80 (0–300)	45.8 (65)	0 (0–100)	0 (0–170)
1st Dx	78.3 (72)	30 (0–100)	60 (0–300)	41.3 (38)	0 (0–70)	0 (0–130)
Relapse	82 (41)	50 (0–100)	95 (0–300)	54 (27)	1 (0–100)	1.75 (0–170)
Bone marrow	79.8 (91)	35 (0–100)	80 (0–300)	49.1 (56)	0 (0–100)	0 (0–170)
1st Dx	80.8 (59)	30 (0–100)	80 (0–300)	42.5 (31)	0 (0–70)	0 (0–130)
Relapse	78 (32)	40 (0–100)	90 (0–300)	61 (25)	1 (0–100)	2 (0–170)
Bone plasmacytoma	71.4 (10)	30 (0–100)	65 (0–300)	35.7 (5)	0 (0–70)	0 (0–90)
1st Dx	60 (6)	7.5 (0–100)	10 (0–265)	30 (3)	0 (0–70)	0 (0–90)
Relapse	100 (4)	65 (30–100)	185 (60–300)	50 (2)	5 (0–10)	5 (0–20)
Hematogenous plasmacytoma	85.7 (12)	20 (0–100)	32.5 (0–297)	28.6 (4)	0 (0–55)	0 (0–75)
1st Dx	77.8 (7)	5 (0–100)	10 (0–101)	44.4 (4)	0 (0–55)	0 (0–75)
Relapse	100 (5)	99 (30–100)	210 (90–297)	0	0	0
ISS stage						
I	90 (9)	7.5 (0–100)	8.5 (0–180)	60 (6)	3.5 (0–70)	4.6 (0–90)
II	73.3 (22)	22.5 (0–100)	40 (0–300)	40 (12)	0 (0–85)	0 (0–130)
III	84.5 (60)	50 (0–100)	90 (0–300)	39.4 (28)	0 (0–100)	0 (0–170)
Ig subtype						
IgG	72.5 (58)	30 (0–100)	70 (0–300)	48.8 (39)	0 (0–100)	0 (0–130)
IgA	95 (19)	50 (0–100)	105 (0–300)	35 (7)	0 (0–20)	0 (0–40)
IgD	100 (1)	100	295	100 (1)	20.5	26.5
Light chain	83.3 (30)	20.5 (0–100)	41.5 (0–300)	50 (18)	3 (0–90)	4 (0–170)
Heavy chain	100 (1)	70	190	0	0	0
Isolated plasmacytoma	100 (2)	72.5 (50–95)	157.5 (50–265)	0	0	0
NA	100 (2)	80 (60–100)	190 (130–250)	0	0	0

### WT1 staining pattern in paired samples

There were 13 cases of bone marrow and plasmacytoma tissues obtained in the same clinical setting. WT1 staining results were concordant in both positivity and staining patterns in most cases, except in two cases with WT1 positivity only in bone marrow samples but negativity in bone plasmacytoma.

There were 22 cases containing sequential samples from the diagnosis and during relapses. Two cases (9%) had no WT1 expression on the diagnosis, then later expressed WT1 at relapse (1 with cytoplasmic staining and 1 with nuclear and cytoplasmic staining). Of 20 cases with WT1 expression at the diagnosis, 17 (85%) cases remained the expression throughout clinical courses. The pattern of WT1 staining was consistent in 11 cases, whereas changes in the staining pattern were found in 6 cases. There were 3 cases with WT1 expression at the diagnosis which turned to be negative at a relapse stage. Interestingly, two of these cases relapsed more than once and became WT1 positive during a later relapse.

### WT1 expression and clinical correlation

To explore the relationship of WT1 expression with clinical characteristics, we analyzed the correlation between WT1 expressions and patients’ clinical parameters. Patients with ISS stage III tended to have a higher percentage of cells with cytoplasmic positivity [10% (0%–100%) vs. 50% (0%–100%), *p* = 0.087] and cytoplasmic H score than those of patients with other stages [11 (0–300) vs. 90 (0–300), *p* = 0.127]. However, the differences were not statistically significant. Other clinical factors, including M protein levels, serum free light chain ratio, hemoglobin, and calcium level, were not statistically different between cases with WT1+ and WT- or cases with different WT1 staining patterns. The summary of clinical parameters in different groups were shown in Table 2 and Supplementary Table S1.

In the patients with positive WT1 staining, the proportion of cells and H-score of cytoplasmic WT1 staining were correlated to high serum free light chain ratio (% positive cells; *p* = 0.011 with a correlation coefficient of 0.284, H score; *p* = 0.039 with correlation coefficient 0.233). No other clinical characteristics (e.g., creatinine, β-2-mg, platelets, ISS staging, etc.) were associated with WT1 expression. The median survival of the patients in this cohort was 45 months. There was no significant association between WT1 expression and treatment response or survival.

## Discussion

In this study, the WT1 expression was investigated by IHC in 142 bone marrow and plasmacytoma samples from a cohort of 95 Thai patients, which is the largest cohort to date. The expression of WT1 in extramedullary MM was first reported in this study. We found that 91.5% of total samples from Thai patients had WT1 expression (Figure 1). High prevalence of WT1 expression was found across all types of tissue samples; bone marrow (92.1%), bone plasmacytoma (78.5%), and hematogenous plasmacytoma (100%). Moreover, a high rate of WT1 expression was still consistent in the samples at the relapse stage (92%; overall relapsed samples, 100%; relapsed plasmacytoma vs. 91.3% in samples at diagnosis). This finding highlights the potential use of WT1 as a target antigen of immunotherapy, particularly in relapsing patients with extramedullary disease that usually respond poorly to currently approved drugs (17). Moreover, the use of WT vaccine to enhance the deeper response during the remission period to control the disease and prevent extramedullary disease is also of interest.

Our results correspond to the high prevalence of WT1 protein expression in MM found in a previous study by Tyler EM et al. (13) despite the different ethnicities of patients. However, this was in contrast to a study from China by Li GJ et al. (16), which showed WT1 expression only in 30% of the patients. In a literature review and our own experience, it has been shown that different WT1 antibody clones and antigen retrieving protocols could offer different staining patterns and positivity rates (4,5,18,19). In our study, similar to Tyler EM et al. (13), the most frequently reported WT1 antibody; clone 6FH7 was used. Unfortunately, we could not access the IHC protocol used by Li GJ et al. (17).

Apart from cytoplasmic WT1 staining reported by Tyler EM et al. (13), which we found in the majority of cases, we also observed nuclear staining of WT1 in a significant number of cases (45.7%). Differences in staining pattern and intensity could be found between sample groups (Figure 2). Extramedullary tissue samples at relapse had a low rate of nuclear WT1 staining, but had a high rate and H-score of cytoplasmic WT1 staining. The cytoplasmic expression of WT1 is more prominent in extramedullary samples compared to in any other tissues analyzed (Figures 2B,C). However, only cytoplasmic WT1 expression has some associations with clinical factors.

The percentage of cells with cytoplasmic WT1 expression and cytoplasmic H-score in samples had positive correlations with a serum free light chain ratio, which could represent a high disease burden and may increase the risk for renal injury (20). However, there was no correlation between WT1 expression and serum creatinine level, rate of kidney dialysis, or treatment outcomes. A further study in a population with more homogenous treatment is needed to clarify the significance of WT1 staining pattern and clinical outcomes.

WT1 gene located on chromosome 11 (Chr 11p13) and transcribed to zinc finger transcription factor that regulates many gene in proliferation and oncogenesis (1). WT1 importance in MM pathogenesis has not been studied. Although chromosome 11 trisomy has been reported in 32.9% of cases in MM genomic landscape study (21), WT1 mRNA overexpression has not been found by gene expression profiling, next generation sequencing or RQ-PCR study (2,11,21,22,23,24). Moreover, WT1 mutation was found only in 0%–0.41% in MM patients (22,23). All these findings suggested that WT1 protein expression found in our study may be correlated with protein translation or post translational regulation of WT1.

WT1 protein is known to have two different functions. Its cellular localization affects the function of the protein (25,26). In the nucleus, WT1 binds to DNA and acts as a transcription factor. It can shuttle to cytoplasm and interact with mRNA, ribonucleoprotein particles (RNPs), and functional polysomes to act as translational regulator (25). Both functions of WT1 as a transcription regulator and a translation regulator may contribute to the pathogenesis of cancer. WT1 has been reported to associate with many oncogenic pathways known in MM pathogenesis. NFκB is one of the main dysregulated pathways in MM pathogenesis (24). The overactivation of NFκB/Rel family members is important for activating the expression of WT1 (27). WT1 expression in the nucleus found in our study may have a transcriptional regulator role, which is known to involve many oncogenic pathways found in MM, e.g., KRAS, MYC, and BCL2 (21,28–31). Interestingly, we found WT1 cytoplasmic expression in the majority of cases. It has been previously shown that WT1 accumulates in the cytoplasm in tumors of different tissue origins (5,32–34). A potential oncogenic role of cytoplasmic WT1 could be the regulation of protein translation. It has been reported that phosphorylation of WT1 by protein kinase A or C causes cytoplasmic retention of WT1 and may decrease the transcription function of WT1 (35). Protein kinase C (PKC) is overexpressed in MM and is important for MM pathogenesis, e.g., cell apoptosis and cell migration (36,37). It is interesting to examine whether PKC activity in MM cells causes WT1 cytoplasmic retention. In our study, extramedullary tissue expressed higher cytoplasmic WT1, especially at relapse in hematogenous plasmacytoma and plasmacytoma. There is evidence that WT1 transcription increases the adhesion molecule and decreases inhibitory chemokine during development (38,39). A further study to clarify whether cytoplasmic retention of WT1 could lead to a decrease in adhesion molecule on MM cells and promote plasmacytoma growth would be of interest.

## Conclusion

Despite low mRNA expression reported in previous studies, we confirmed that the high prevalence of WT1 protein expression detected by IHC could be found in the MM samples. We have shown for the first time that WT1 has high expression rates in both medullary myeloma and plasmacytoma regardless of disease status. There was an exceptionally high rate of WT1 expression in the cases with hematogenous plasmacytoma and plasmacytoma at the relapse stage, which were difficult to treat. Our findings support the use of WT1 as a target antigen for immunotherapy in MM and plasmacytoma irrespective of disease status. WT1 expression detected by IHC could be a potential marker for WT1 immunotherapy; however, antibody and IHC protocol might affect WT1 positive rate and staining pattern. WT1 correlated with a high-risk clinical feature but it did not correlate with poor outcome.

## Data Availability

The original contributions presented in the study are included in the article/Supplementary Material, further inquiries can be directed to the corresponding authors.
