# Supplementary material for: High prevalence of Wilms tumor 1 expression in multiple myeloma and plasmacytoma: A cohort of 142 Asian patients’ samples
Source: Pathol Oncol Res. 2023 Jan 24;29:1610844. doi: 10.3389/pore.2023.1610844 (PMC9902379; doi:10.3389/pore.2023.1610844)
Supplement: Supplementary file 1 [file Table1.DOCX]

**Supplementary table 1**. clinical parameters in correlation with WT1 staining pattern

|  | **Negative** | **Exclusively cytoplasmic staining** | **Exclusively nuclear staining** | **Both staining** | **P value** |
| --- | --- | --- | --- | --- | --- |
| **Hemoglobin (g/dl)** | 8.6 ± 2 | 8.7 ± 2.2 | 8.7 ± 2.5 | 9.2 ± 1.9 | 0.568 |
| **Creatinine (mg/dl)** | 1.19  (0.54-2.25) | 1.16  (0.56-7.78) | 1.19  (0.62-7.02) | 1.42  (0.36-15.4) | 0.904 |
| **Calcium (g/dl)** | 10.6 ± 1.2 | 10.2 ± 1.2 | 10.2 ± 1.8 | 10.6 ± 2.3 | 0.602 |
| **Beta2 microglobulin (mg/dl)** | 6.3  (3-12.5) | 7.5  (1.9-47.8) | 5.9  (3.4-19.7) | 6.2  (1.8-69.1) | 0.648 |
| **M-protein (g/dl)** | 4.51  (2.47-6.13) | 4.4  (0.26-7.74) | 5.1  (1.83-6.24) | 3.25  (0.34-6.55) | 0.075 |
| **Serum free light chain ratio** | 8.02 (5.56-241.72) | 67.2 (1.05-5537.8) | 184.8 (2.16-386.8) | 160.8 (1.14-1171.4) | 0.131 |
| **ISS stage (%, n)**  **I**  **II**  **III** | 10% (1)  10% (3)  8.5% (6) | 30% (3)  50% (15)  52.1% (37) | 0  16.7% (5)  7% (5) | 60% (6)  23.3% (7)  32.4% (23) | 0.303 |
| **Subtype (%, n)**  **IgG**  **IgA**  **IgD**  **Light chain**  **Heavy chain**  **Isolated plasmacytoma**  **NA** | 11.3% (9)  5% (1)  0  5.6% (2)  0  0  0 | 40% (32)  60% (12)  0  44.4% (16)  100% (1)  100% (2)  100% (2) | 16.3% (13)  0  0  11.1% (4)  0  0  0 | 32.5% (26)  35% (7)  100% (1)  38.9% (14)  0  0  0 | 0.682 |
